# Bottom-Up Effects of Dietary Cadmium Exposure on Asian Corn Borer *Ostrinia furnacalis* and Its Egg Parasitoid *Trichogramma ostriniae*: Bioaccumulation and Biocontrol Risks

**DOI:** 10.3390/insects17080828

**Published:** 2026-08-10

**Authors:** Jie Wang, Cheng-Xing Wang, Yan Li, Nicolas Desneux, Su Wang, Bin Tang

**Affiliations:** 1 College of Life and Environmental Sciences, Hangzhou Normal University, Hangzhou 311121, China; jlyk@hznu.edu.cn; 2 Institute of Plant Protection, Shandong Academy of Agricultural Sciences, Jinan 250131, China; 15006410568@163.com; 3 Université Côte d’Azur, INRAE, UMR-ISA, 06000 Nice, France; nicolas.desneux@inrae.fr; 4 Key Laboratory of Natural Enemies Insects, Ministry of Agriculture and Rural Affairs, Institute of Plant Protection, Beijing Academy of Agriculture and Forestry Sciences, Beijing 100097, China; wangsu@baafs.net.cn

**Keywords:** heavy metal pollution, cadmium, *Ostrinia furnacalis*, biological control, bottom-up effect

## Abstract

Cadmium (Cd) is a pervasive heavy metal pollutant in agricultural ecosystems, posing toxic threats to crop productivity, food safety, and the efficacy of biological control programs. The Asian corn borer (ACB), *Ostrinia furnacalis* is a destructive lepidopteran pest of maize in East Asia, while *Trichogramma ostriniae* serves as the most widely applied egg parasitoid for the sustainable management of this pest in integrated pest management (IPM) programs. However, the cascading effects of sublethal Cd stress transmitted from herbivorous hosts to egg parasitoids remain poorly understood. Here, we investigated the sublethal effects of dietary Cd exposure (5.0 mg/kg) on the development and Cd bioaccumulation of ACB, and subsequently evaluated the parasitism performance of the egg parasitoid *T. ostriniae* reared on Cd-contaminated host eggs. Results showed that chronic Cd exposure did not significantly affect larval weight, male pupal weight, or female pupal weight of ACB, but significantly increased female adult body weight, indicating stage-specific stimulatory effects. Cd concentrations in larvae, female pupae, and female adults of ACB were markedly elevated following dietary exposure, with the highest Cd loads detected in female pupae (41.23 mg/kg). However, maternal Cd transfer to eggs did not result in a statistically significant increase in egg Cd concentration. Notably, parasitism by *T. ostriniae* on Cd-contaminated ACB eggs significantly increased the number of eggs parasitized, although offspring emergence rate and female progeny ratio remained unaffected. This study fills the research gap regarding the trophic transfer of Cd between ACB and *T. ostriniae*, clarifies the complex bottom-up effects of Cd pollution on host–parasitoid interactions, and provides empirical references for the rational deployment of *T. ostriniae* as a biocontrol agent in Cd-contaminated maize fields.

## Simple Summary

Heavy metals prevalent in agricultural soils can transfer through food chains and interfere with pest biological control. Here, we investigated the bioaccumulation of sublethal cadmium (5.0 mg/kg) in different life stages of the Asian corn borer (ACB), *Ostrinia furnacalis* (Guenée) (Lepidoptera: Pyralidae), and its bottom-up effects on the parasitism performance of the dominant egg parasitoid *Trichogramma ostriniae* (Pang et Chen) (Hymenoptera: Trichogrammatidae). Our results showed that Cd was highly accumulated in larvae, female pupae and female adults of ACB, while no obvious Cd enrichment was detected in host eggs. Sublethal Cd exposure did not affect the body weight of ACB larvae and pupae, but significantly increased the body mass of female adults. Host Cd contamination markedly raised the number of eggs parasitized by *T. ostriniae*, whereas parasitoid emergence rate and female progeny ratio remained unchanged. This study demonstrates that sublethal Cd stress causes stage-specific bioaccumulation and physiological alterations in ACB, and exerts a stimulatory effect on the parasitism capacity of *T. ostriniae* without impairing its developmental fitness. Our findings provide practical references for the application of *T. ostriniae* as a biocontrol agent in Cd-polluted maize fields.

## 1. Introduction

Heavy metal contamination in soil has become an increasingly serious environmental concern in agricultural ecosystems worldwide, largely due to anthropogenic activities such as mining, sewage irrigation, industrial emissions, and excessive application of metal-containing agrochemicals [1]. Among toxic heavy metals, cadmium (Cd) is of particular concern because of its high toxicity, mobility, and persistence in soils, with a biological half-life exceeding several decades [2,3]. A global machine learning modeling assessment covering worldwide agricultural zones estimated that 14–17% of global cropland exceeds agricultural thresholds for at least one toxic metal, with Cd ranking among the most prevalent soil contaminants across this global dataset [1]. Unlike other pollutants, Cd can continuously migrate from soil to crops, then to herbivorous insects, predators and parasitoids via trophic cascades, thereby disrupting the structure and function of agroecosystems and weakening natural pest suppression services [4,5,6,7].

In China, intensive agricultural production and rapid industrialization have exacerbated soil Cd pollution, especially in maize-planting areas [2,8]. Maize (*Zea mays* L.) is a staple crop cultivated worldwide and has been widely adopted for combined agricultural production and phytoremediation practices on Cd-polluted farmland across China [3,9,10]. Field surveys have confirmed that Cd concentrations in maize rhizosphere soil and maize tissues frequently exceed national safety standards in many regions, and Cd can be readily absorbed and enriched by maize roots and transported to aboveground tissues [11,12]. Cd-contaminated maize not only threatens food security and human health through dietary intake but also acts as a pollution source to transmit Cd to insect herbivores feeding on maize, initiating a series of biological responses along the plant–insect-natural enemy food chain [3,13]. Previous studies have verified that Cd stress can alter the nutritional composition and defensive metabolites of maize, further changing the growth, development and reproduction of herbivorous pests [3,14].

The Asian corn borer (ACB), *Ostrinia furnacalis* (Guenée) (Lepidoptera: Pyralidae), ranks as Asia’s most destructive maize pest. Its feeding injuries to corn leaves, stalks and ears trigger annual grain losses ranging from six to nine million tons solely within China [15,16,17]. Previous studies have demonstrated that ACB larvae can accumulate Cd from contaminated artificial diets or host plants, and that Cd exposure affects ACB development, mating behavior, and fecundity [18,19]. Specifically, Cd exposure has been shown to extend larval and pupal duration, reduce survival rates, and impair reproductive behaviors, with residual effects persisting into adulthood even when exposure occurs only during early life stages [18]. Furthermore, ACB larvae primarily accumulate Cd in the digestive tract, and Cd concentrations gradually decrease from larval to pupal to adult stages [18]. However, the effects of Cd on ACB have been investigated primarily using single concentrations, and dose-dependent responses remain insufficiently characterized.

Egg parasitoids from the genus *Trichogramma* (Hymenoptera: Trichogrammatidae) stand as one of the globally dominant biological control agents. These parasitoids are broadly deployed to suppress lepidopteran pests in agricultural and forestry systems [20,21]. As typical egg parasitoids of the genus *Trichogramma*, multiple species have been artificially mass-reared and released on a large scale in China since the 1970s, becoming the cornerstone of integrated pest management (IPM) strategies for major agricultural pests [22,23,24]. Among them, *Trichogramma ostriniae* (Pang et Chen) is recognized as the most efficient biocontrol agent against ACB, with higher parasitism rate, stronger host adaptability and better multi-generational stability compared with *T. dendrolimi* (Matsumura) and *T. chilonis* (Ishii) [15,16,25]. Field monitoring data show that the parasitism rate of *T. ostriniae* on ACB eggs can reach up to 90% during pest outbreak periods, achieving pest control efficacy equivalent to chemical pesticides while maintaining ecological diversity [25].

Nevertheless, the widespread Cd pollution in farmland inevitably threatens the fitness and biocontrol efficiency of parasitoid wasps via bottom-up trophic effects [26]. Recent studies have explored the effects of Cd on different *Trichogramma* species using factitious hosts such as rice moth [*Corcyra cephalonica* (Stainton) (Lepidoptera: Pyralidae)]. The results show that Cd stress induces species-specific responses in *Trichogramma*, where high-dose Cd significantly reduces the survival rate, longevity and female ratio of *T. dendrolimi*, while *T. ostriniae* and *T. japonicum* Ashmead exhibit relatively strong tolerance to Cd-contaminated food resources [27]. Cadmium exposure also triggers transgenerational effects and changes host preference of *T. dendrolimi* and *T. japonicum*, further complicating host–parasitoid interactions in polluted fields [28]. In addition, Cd can be transmitted to parasitoids through host pupae, leading to oxidative damage, energy metabolism disorder and reduced parasitic fitness of parasitoid offspring [29,30]. Although previous studies mostly focus on high-concentration lethal Cd stress [18,31], the ecological impacts of sublethal Cd concentrations on host bioaccumulation and parasitoid performance are rarely reported. It remains unclear whether Cd accumulated in different developmental stages of ACB can be stably transmitted to eggs, and how such changes in host egg quality affect the key parasitism traits of *T. ostriniae* [4,5].

To address these knowledge gaps, the present study was designed to: (i) evaluate the sublethal effects of dietary Cd exposure at a representative concentration (5.0 mg/kg) on the development and Cd bioaccumulation of ACB across different life stages; (ii) quantify Cd transfer from the herbivore to its eggs; and (iii) assess the parasitism performance (number of eggs parasitized, emergence rate, and female adult ratio) of *T. ostriniae* reared on Cd-contaminated ACB eggs. By integrating these analyses, this study aims to provide a more comprehensive understanding of how Cd contamination cascades through the herbivore (ACB)–parasitoid (*T. ostriniae*) food chain and to generate evidence-based recommendations for the deployment of biological control agents in Cd-contaminated agricultural landscapes.

## 2. Material and Methods

### 2.1. Insects Rearing

The Asian corn borer eggs were from Jilin Academy of Agricultural Sciences (Changchun, China). The Asian corn borer colony was maintained in a rearing room under controlled conditions (26 ± 1 °C, 60 ± 5% RH). Larvae were reared on artificial diet in plastic boxes (22.7 cm × 14.8 cm × 7.7 cm). After emerging, the adults were paired in nylon cages (40 cm × 40 cm × 75 cm) and provided with 20% (*v*/*v*) honey solution. Butter papers were provided for the eggs laid [15].

The egg parasitoid, *Trichogramma ostriniae*, originated from Lab of Insect Molecular Ecology and Evolution of Nanjing Agricultural University, China. Fresh rice moth eggs inactivated under ultraviolet light for 30 min were provided for parasitism. Parasitoids were reared under laboratory conditions (26 ± 1 °C, 80 ± 5% RH, 16L:8D). Adult wasps were provided with a 10% (*w*/*v*) sucrose solution as a carbohydrate source [32].

### 2.2. Development of the Asian Corn Borer Under Cadmium Exposure

A previous study has measured maize Cd concentrations across treatments. Its values fell between 0.02 and 0.07 mg/kg, where Cd level represented a sublethal concentration for ACB [31]. Luo et al. [18] tested a 5 mg/kg sublethal Cd dose in artificial diets. ACB larvae fed this diet remained viable. This dose generates sufficient insect numbers for subsequent trials. The 5.0 mg/kg concentration in artificial diet is not intended to replicate the bulk tissue Cd content of field-grown maize, but rather to deliver a biologically effective sublethal dose that produces measurable physiological effects in ACB while maintaining larval viability. Moreover, this concentration falls within the range commonly used in artificial diet studies of lepidopteran insects (typically 1–10 mg/kg) to ensure adequate metal bioavailability and detectable toxicological effects under controlled laboratory conditions [33,34,35]. We adopted 5.0 mg/kg sublethal Cd in the present work to examine how Cd exposure alters the fitness of ACB and *T. ostriniae*.

Cadmium chloride (CdCl_2_, 99.8% purity, Shanghai Aladdin Bio-Chem Technology Co., LTD, Shanghai, China) served as the cadmium source in this study. The chemical powder was dissolved in distilled water before use. The artificial diet for ACB larvae was prepared as per Wang et al. [15]. The basic diet mixture consisted of 300 g wheat bran, 100 g yeast, 8 g methyl 4-hydroxybenzoate, 8 g sorbic acid, 8 g ascorbic acid, 50 µL linoleic acid, 28 g sucrose, 30 g agar, and 1500 mL distilled water. To establish the Cd treatment group, 10.0 mL of prepared Cd solution was blended evenly into the artificial diet, while an equal volume of distilled water was supplemented into the control diet.

Larval rearing was conducted in plastic containers (15.7 cm × 11.0 cm × 7.7 cm) supplied with the prepared artificial diet. In each container, 150 newly hatched, synchronous neonate larvae were induced for rearing. Each container represented one biological replication, with three replications for control group and the 5.0 mg/kg Cd treatment group, separately (*N* = 3). Upon pupation, all individuals were collected and transferred into a mesh cage (40 cm × 40 cm × 75 cm) for adult emergence. After eclosion, adult moths were fed with a 20% (*v*/*v*) honey solution dispensed on cotton wicks, following the standard rearing protocol for ACB adults reported by Wang et al. [15]. A 40 cm × 40 cm sheet of butter paper was fixed inside each cage as oviposition substrate, which was regularly replaced to collect newly deposited eggs throughout the adult stage [15]. Among these, individual insects were randomly sampled across the three replicate containers, with single individuals treated as the statistical experimental unit. Sample sizes varied markedly across developmental stages due to differences in insect availability and subsequent experimental demands. Abundant larvae and pupae were readily harvested from replicate containers, enabling sampling of 60 larvae after exposure to Cd for 10 days (*N* = 60), 50 male pupae (*N* = 50) and 50 female pupae (*N* = 50) for weight detection. However, emerged female adults were limited in total quantity, and most female moths were retained to produce fresh ACB eggs for the follow-up parasitism performance test of *T. ostriniae*. To guarantee sufficient host egg supply for parasitoid bioassays, only 10 female adults (*N* = 10) were randomly selected and sacrificed for body weight measurement. Weights were weighted by the balance (XPR404S, Mettler Toledo, Greifensee, Switzerland).

### 2.3. Cadmium Content Detection in the Asian Corn Borer

Three larvae after 10 d Cd exposure, two-day-old female pupae, two-day-old female adults and about 1500 eggs from each replication were randomly collected from each replicate of the control and 5.0 mg/kg Cd exposure groups. Larval and pupal samples were rinsed three times with distilled water to eliminate residual dietary contaminants that could interfere with Cd quantification. Notably, five replicates were prepared for egg Cd detection instead of three replicates used for larval, pupal and adult samples from Cd treatment and control. The eggs showed strong inherent heterogeneity in Cd residue distribution; preliminary trials confirmed that three replicates produced high measurement variability. Extra egg replicates were therefore set to lower analytical error and strengthen the robustness of statistical comparison, whereas larvae, female pupae and female adults exhibited more uniform Cd bioaccumulation within treatments, making three biological replicates adequate for reliable quantification. All samples were collected and preserved at −20 °C, followed by transfer into plastic Petri dishes with a diameter of 10 cm. Samples were oven-dried at 65 °C for a minimum of 48 h until a constant dry weight was achieved. The dried specimens were then ground into fine homogeneous powder for detection. Cadmium quantification was completed by Pony Testing International Group (China), a qualified third-party professional analytical laboratory with certified heavy metal detection capabilities. For sample pretreatment, 0.20 g of powdered sample was weighed and placed into the inner vessel of a microwave digestion system. For samples containing ethanol or carbon dioxide, low-temperature preheating on an electric hot plate was performed in advance to remove residual organic solvents and gases. A volume of 5 mL of nitric acid was added to the vessel, which was then loosely covered and kept stationary 1 h before tight-capping. The solution was diluted to a constant volume of 25 mL with water and mixed well for subsequent analysis. A reagent blank sample was prepared synchronously throughout the whole procedure to eliminate background interference. The Cd concentrations in all samples were determined via Inductively Coupled Plasma–Mass Spectrometry (ICP-MS) (7700, Agilent Technologies, Santa Clara, CA, USA). Strict quality assurance and quality control (QA/QC) protocols were implemented throughout Cd quantification. A standard biologically certified reference material (CRM, GBW10012) was tested alongside all insect samples to validate measurement accuracy. The Cd recovery rate of CRM ranged from 92.4% to 104.7%, complying with the acceptable 80–120% recovery standard for heavy metal analysis. The method detection limit (MDL) of Cd via ICP-MS was 0.0012 μg L^−1^. Reagent blanks were digested and measured in parallel for every 10 samples to eliminate contamination from acids and digestion vessels. Each biological sample was measured with technical duplicates, and relative standard deviation (RSD) of duplicate readings was controlled below 5% to guarantee instrumental precision.

### 2.4. Parasitism Performance of Trichogramma ostriniae Under Cadmium Exposure

**Figure 1:**
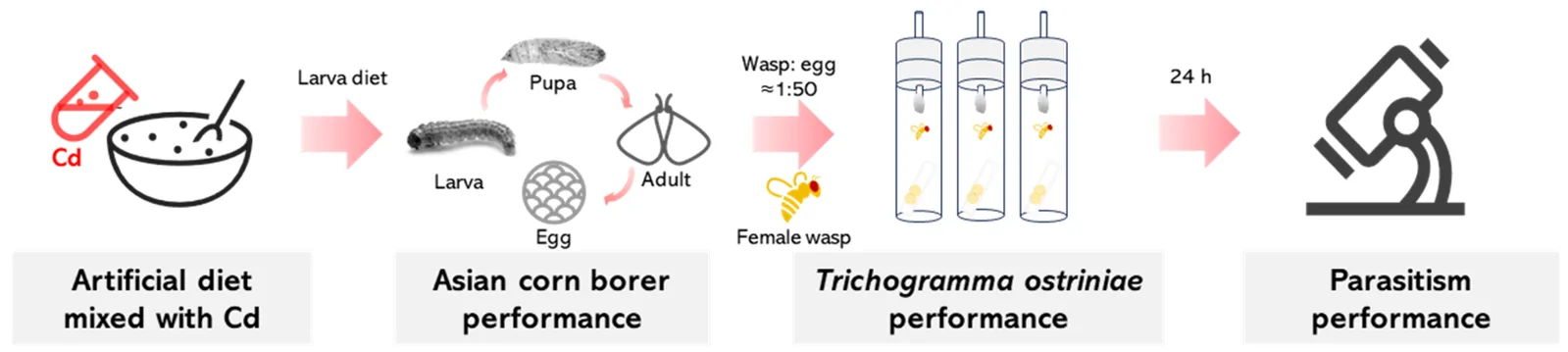
Schematic diagram of the effect of cadmium exposure on the Asian corn borer and *Trichogramma* wasp performance.

Female wasps emerging from rice moth eggs mated within 8 h of eclosion and were used for parasitism performance bioassays [15,20,21]. Fresh ACB eggs (<24 h) were attached in a single layer with double-sided tape to a paper card (50–70 eggs per card, 3.0 cm long × 2.0 cm wide), following the bioassay protocol of Wang et al. [15]. This egg number surpasses the maximum daily parasitism capacity of one mated female wasp to guarantee non-limiting host availability during the assay. Each card was placed in a plastic tube (3.0 cm diameter × 10.0 cm height), and a single mated female wasp was introduced. Tubes were plugged with cotton-stoppered foam plugs, and 0.1 mL of 10% (*w*/*v*) sucrose solution was supplied as adult nutrition (Figure 1). All tubes were incubated at 25 ± 1 °C, 70% RH, in a climate chamber (PGX-350D, Ningbo Saifu Laboratory Instrument Co., Ltd., Ningbo, China). Each tube represented one replicate, with 22 and 41 replicates for control and Cd treatment, respectively. The unbalanced sample sizes were designed to improve statistical power: preliminary observations revealed higher inter-individual variability in parasitism capacity among wasps exposed to Cd-contaminated host eggs, hence extra replicates were allocated to the Cd treatment to minimize random error. In contrast, parasitism metrics of wasps provided with untreated control eggs displayed low variation, so 22 replicates were adequate for reliable statistical comparison. All replicates for both groups were maintained under identical incubation conditions throughout the assay.

Wasps were removed after 24 h of exposure to host eggs. Host egg cards were incubated under the same condition. After all progeny wasps had emerged, black parasitized eggs (number of parasitized eggs) and emergence holes (number of emerged parasitized eggs) were counted under a stereomicroscope (Stemi 508, Zeiss, Oberkochen, Germany). Females and males were distinguished by antennal morphology. Life-history parameters were calculated as follows: female adult ratio (%) = (number of emerged females/number of emerged adults) 100, and emergence rate (%) = (number of emerged parasitized eggs/number of parasitized eggs) 100.

### 2.5. Statistical Analysis

All statistical analyses were performed with GraphPad Prism 9.0.0 (GraphPad LLC, Boston, MA, USA). Prior to formal data comparison, the Shapiro–Wilk test and the Brown–Forsythe test was applied sequentially to assess normality and homogeneity of variance, respectively. For ACB body weight metrics (larval, male pupal, female pupal, female adult weight), the data satisfied both normality and homoscedasticity assumptions, so two-tailed unpaired Student’s *t*-tests were used to compare control and Cd-treated groups (*p* < 0.05). For whole-body Cd concentration data across developmental stages, the data deviated from normal distribution; thus, non-parametric Mann–Whitney *U* tests were adopted for pairwise comparisons between treatments (*p* < 0.05). For parasitism-related parameters (number of parasitized eggs, parasitoid emergence rate, female progeny ratio), these datasets also failed normality screening, and group differences were evaluated using Mann–Whitney *U* tests (*p* < 0.05). All results are presented as means ± standard error (SE). Statistical significance was defined at *p* < 0.05.

## 3. Results

### 3.1. Effect of Cadmium Exposure on the Development of the Asian Corn Borer

**Figure 2:**
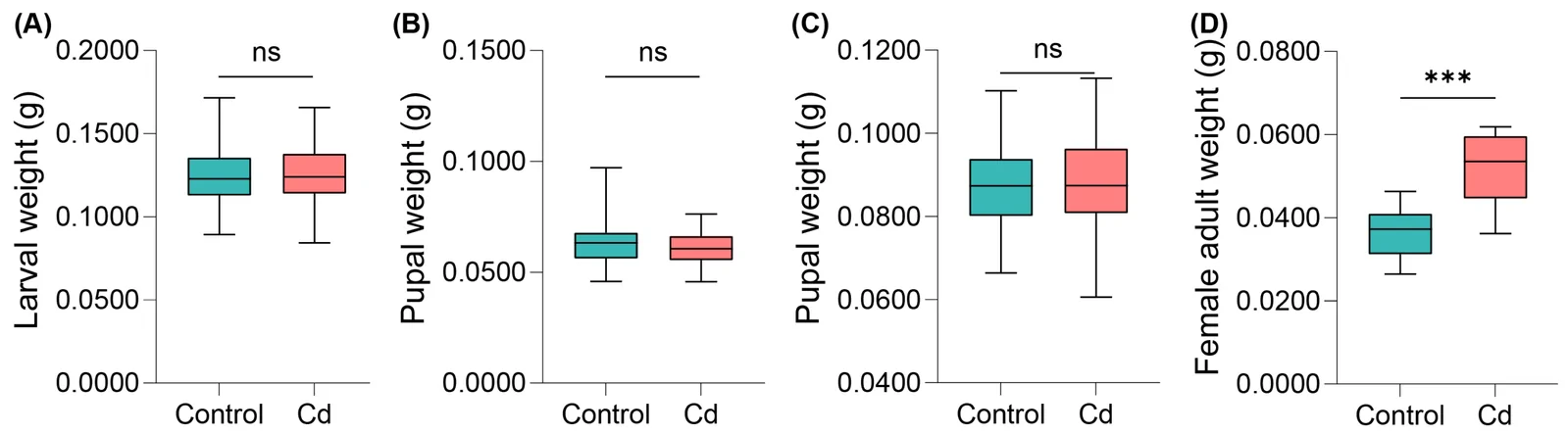
Effect of cadmium sublethal exposure (5.0 mg/kg) on the Asian corn borer performance. The larval weight after exposure to Cd for 10 days (**A**), the male pupa weight (**B**), the female pupa weight (**C**), and female adult weight (**D**). Values are given as means ± SE. Significance was determined by the two-tailed unpaired Student’s *t* test (ns, *p* > 0.05; ***, *p* < 0.001).

Chronic dietary exposure for 10 days to 5.0 mg/kg Cd exerted no significant effects on larval weight (Figure 2A; control mean: 0.1250 g, Cd treatment mean: 0.1250 g; *t* = 0.183, *df* = 118, *p* = 0.8550), male pupal weight (Figure 2B; control mean: 0.0631 g, Cd treatment mean: 0.0607 g; *t* = 0.183, *df* = 98, *p* = 0.8555), or female pupal weight (Figure 2C; control mean: 0.0841 g, Cd treatment mean: 0.0875 g; *t* = 1.40, *df* = 98, *p* = 0.1634) of ACB. Mean body masses were nearly identical between the Cd-treated and control groups at these developmental stages, accompanied by very small eta-squared effect sizes indicating minimal biological perturbation. By contrast, Cd exposure significantly increased adult female body weight (Figure 2D, *t* = 4.62, *df* = 18, *p* = 0.0002); the average weight of Cd-exposed females (0.0521 g) was markedly higher than that of control females (0.0366 g). This represents a distinct stage-specific response to sublethal Cd exposure in the Asian corn borer. Notably, this pattern was observed under only one tested Cd concentration, and further multi-concentration trials are required to confirm whether this biomass increase reflects a consistent hormetic stimulatory response across pollution gradients.

### 3.2. Cadmium Contamination on the Asian Corn Borer

Larval Cd concentration reached 38.07 ± 3.22 mg/kg in the Cd treatment group, markedly higher than the control level of 0.08 ± 0.01 mg/kg (Table 1, *p* < 0.05). Similarly, female pupae from Cd exposure accumulated substantially greater Cd (41.23 mg/kg) compared with control female pupae (0.11 mg/kg, Table 1, *p* < 0.05). Cd residues remained significantly higher in Cd-treated female adults (3.31 mg/kg) than control female adults (0.04 mg/kg, Table 1, *p* < 0.05). In contrast, egg Cd concentrations showed no statistically significant difference between the control (0.05 ± 0.01 mg/kg) and Cd treatment (0.19 ± 0.09 mg/kg) groups (Table 1, *p* > 0.05). Overall, dietary Cd exposure induced pronounced bioaccumulation in larval, female pupal and female adult stages of ACB, with the highest Cd loads observed in female pupae, whereas maternal Cd transfer into eggs did not produce a statistically significant elevation in egg Cd concentration under this exposure regime.

### 3.3. Effect of Cadmium Exposure on the Parasitism Performance of Trichogramma ostriniae

**Figure 3:**
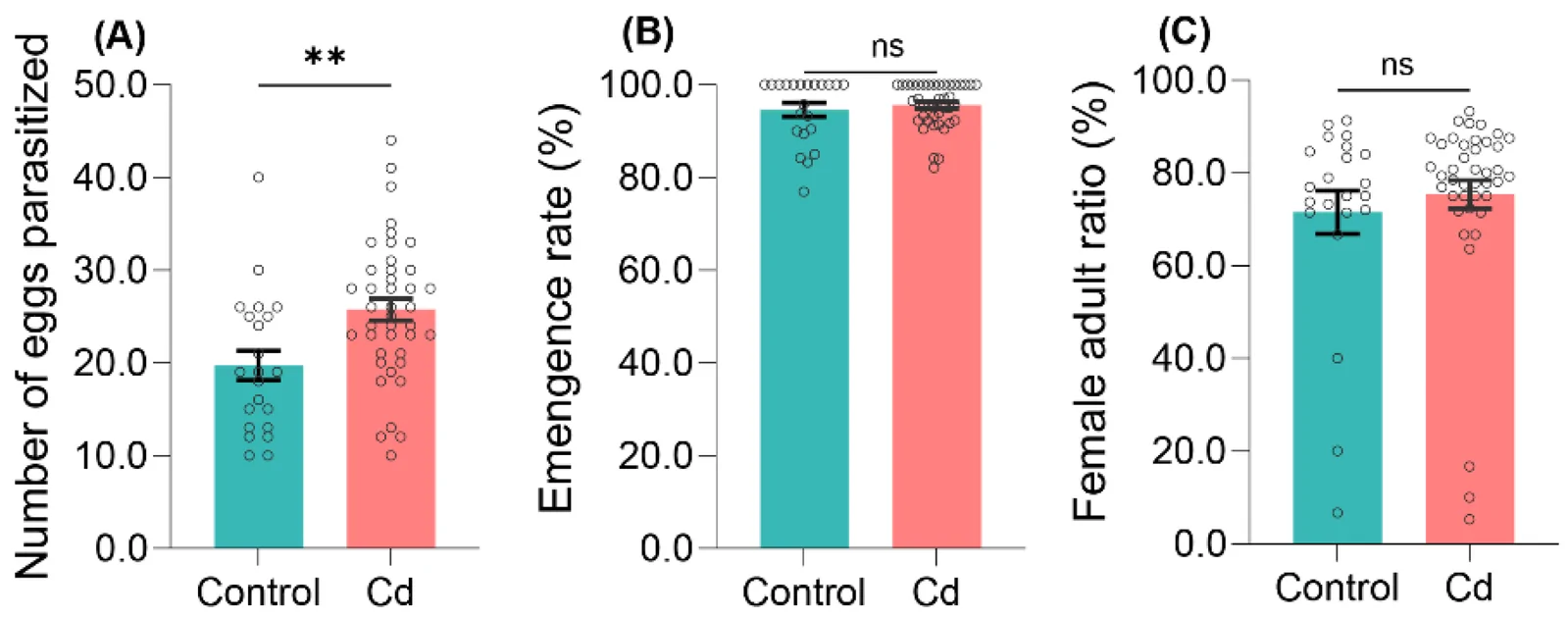
Parasitism performance of *Trichogramma ostriniae* under cadmium sublethal exposure (5.0 mg/kg). Number of parasitized eggs (**A**), emergence rate (**B**), and female adult ratio (**C**) of *T. ostriniae*. Values are given as means ± SE. Significance was determined by the Mann–Whitney *U* test (ns, *p* > 0.05; **, *p* < 0.01).

Host cadmium contamination significantly elevated the number of eggs parasitized by *T. ostriniae* (Figure 3A, *U* = 249, *p* = 0.0031), with median parasitism rising from 19.0 (control) to 26.0 (Cd treatment). However, neither offspring emergence rate (Figure 3B, *U* = 446, *p* = 0.9432) nor female adult ratio (Figure 3C, *U* = 380, *p* = 0.3100) differed significantly between treatments, indicating that Cd-mediated host exposure only stimulated parasitism quantity without altering wasp developmental fitness or sex allocation.

## 4. Discussion

The present study provides novel insights into the bottom-up effects of sublethal cadmium (Cd) exposure along the food chain of ACB, and its egg parasitoid *T. ostriniae*. Our results demonstrate that dietary Cd exposure at 5.0 mg/kg produced stage-specific effects on ACB development, significantly increasing female adult body weight without affecting larval or pupal weights. Moreover, Cd bioaccumulated markedly in ACB larvae, female pupae, and female adults, but maternal transfer to eggs was not statistically significant. Parasitism by *T. ostriniae* on Cd-contaminated ACB eggs was significantly enhanced in terms of the number of eggs parasitized, while emergence rate and female progeny ratio remained unaffected. These findings have important implications for understanding the ecological consequences of heavy metal pollution on biological control services in agricultural ecosystems.

Contrary to our expectation that Cd exposure would universally impair ACB growth, we observed that chronic dietary Cd at 5.0 mg/kg did not significantly affect larval weight, male pupal weight, or female pupal weight, but significantly increased female adult body weight. This stage-specific stimulatory effect, observed only in adult females, is suggestive of hormesis-like response, a biphasic dose–response phenomenon in which low or sublethal doses of a toxicant produce beneficial or stimulatory effects [36,37]. However, we acknowledge that the single concentration used in this study does not permit definitive confirmation of a hormetic dose–response relationship. Hormetic responses to heavy metals have been documented in several herbivorous insects. For example, low concentrations of Cd (0.30 and 0.60 mg/kg) increased larval body mass of the oriental armyworm, *Mythimna separata* (Walker) (Lepidoptera: Noctuidae), whereas higher concentrations caused inhibition [33]. However, pupal weight declined under the same Cd concentrations, and sex-specific effects on body weight were not reported in that study [33]. Similarly, low doses of Cd promoted population growth of the beet armyworm, *Spodoptera exigua* (Hübner) (Lepidoptera: Noctuidae), while high doses suppressed it, although weight measurements were not analyzed separately for males and females [38]. Notably, the stage- and sex-specific stimulation observed in our study, whereby only female adults exhibited increased body mass, adds a new dimension to the understanding of hormetic responses in lepidopteran herbivores under heavy metal stress, highlighting that Cd effects can be highly selective across developmental stages and between sexes. In spider mites, Cd-accumulating tomato plants induced a non-linear, hormetic response in some populations [39]. The stimulatory effect on female adult weight in our study may reflect an adaptive response whereby ACB females allocate additional resources to body mass under mild Cd stress, potentially as a compensatory mechanism to maintain reproductive output despite the costs of detoxification [33]. The adaptive strategies employed by insects under heavy metal stress are multifaceted and operate at multiple levels of biological organization. At the physiological level, insects can upregulate detoxification enzymes—such as glutathione S-transferases (GSTs), carboxylesterases (CarEs), and cytochrome P450 monooxygenases—to metabolize and eliminate accumulated metals [40]. At the molecular level, metal-binding proteins such as metallothioneins (MTs) play a critical role in sequestering excess Cd and maintaining heavy metal homeostasis [41]. However, it is important to note that increased adult weight does not necessarily translate into enhanced fitness, as previous studies have shown that Cd exposure can impair mating behavior and reduce fecundity even when body size is unaffected or increased [18]. Elucidating the relative contributions of these adaptive mechanisms, and their associated costs, remains a critical direction for future research on insect responses to heavy metal pollution in agricultural landscapes.

The absence of significant effects on larval and pupal weights in our study contrasts with some previous reports. For instance, Luo et al. [18] reported that Cd exposure at 5 mg/kg extended larval and pupal duration and decreased survival rates of ACB, but they did not report weight parameters. In other lepidopterans, Cd has been shown to reduce pupal weight in *M. separata* at intermediate and high concentrations [33] and to decrease larval and pupal weights in the tobacco cutworm, *Spodoptera litura* (Fabricius) (Lepidoptera: Noctuidae) [34]. Conversely, some studies have found no effect of Cd on certain developmental parameters. For example, Huang et al. [35] reported that Cd exposure did not affect larval duration or pupation rate of the rice striped stem borer, *Chilo suppressalis* (Walker) (Lepidoptera: Crambidae), although pupal weight was reduced at high Cd levels (5.0 and 10.0 mg/kg). These discrepancies may be attributed to differences in exposure duration, Cd concentration, insect species, and the specific endpoints measured. Our study used a concentration of 5.0 mg/kg, which is within the sublethal range for ACB [18], and we measured weights at specific time points rather than developmental duration or survival. The stage-specific nature of Cd effects underscores the importance of assessing multiple fitness parameters across the entire life cycle when evaluating pollutant impacts.

In terms of Cd bioaccumulation, our results verified that Cd can be continuously enriched in larvae, female pupae and female adults of ACB, and female pupae are the key stage for Cd accumulation, which is partially consistent with the conclusion of Luo et al. [18] that Cd concentration gradually decreases from larvae to adults. ACB larvae efficiently accumulate Cd from their food, which is consistent with previous reports on ACB [18] and other lepidopterans such as the greater wax moth, *Galleria mellonella* Linnaeus (Lepidoptera: Pyralidae) [42] and *S. exigua* [38]. A striking bioaccumulation pattern observed here is the drastic decline in whole-body Cd levels from female pupae (41.23 mg/kg) to newly emerged female adults (3.31 mg/kg), with over 90% Cd lost during metamorphosis. This sharp depletion represents a vital adaptive trait of ACB against Cd toxicity and vertical metal transmission to eggs. Most Cd absorbed by larvae accumulates in midgut epithelia, which undergoes complete histolysis during pupal development [18]. The majority of sequestered Cd is excreted via meconium upon adult eclosion, instead of being redistributed into adult reproductive tissues [40]. This metamorphic metal clearance delivers dual fitness benefits. This physiological mechanism jointly protects maternal adults from heavy metal poisoning and shields progeny from excessive Cd exposure, revealing a critical stage-specific detoxification strategy in lepidopterans to cope with persistent soil metal pollution.

Notably, our study found that the Cd concentration in ACB eggs had no significant difference between the control and Cd treatment groups. This differs from the research on *C. suppressalis* [35], where Cd in parental larvae was successfully transmitted to eggs. We infer that the maternal transfer efficiency of Cd varies among different lepidopteran species, and ACB may have a certain barrier mechanism to prevent excess Cd from being transferred to eggs during oviposition, which protects the next generation of larvae from direct Cd damage [43]. However, this interpretation remains speculative, as our experimental design does not directly demonstrate such a mechanism. The egg Cd concentration was 0.08 ± 0.04 mg/kg in the control group and 0.19 ± 0.09 mg/kg under sublethal Cd exposure (5.0 mg/kg), showing considerable overlap driven by substantial inter-replicate variability that may mask subtle inter-treatment differences. Notably, all egg replicates were randomly sampled, processed uniformly, and screened for outliers prior to analysis; no abnormal replicates were identified or excluded from the dataset. This variability could reflect individual differences in maternal Cd allocation or stochastic variation during oviposition [18]. Cadmium dynamics during reproduction may involve differential partitioning among reproductive tissues (ovaries, accessory glands, mature eggs), with only a fraction of maternal Cd ultimately deposited into laid eggs [44]. While this may benefit ACB offspring, it carries significant ecological risks. First, Cd retention in maternal tissues, particularly at high concentrations in female pupae (41.23 mg/kg) and adults (3.31 mg/kg), implies that females bear the toxic burden, potentially reducing longevity, flight capacity, or dispersal behavior [18]. Second, even without elevated egg Cd, maternal stress can induce epigenetic changes or alter egg quality (lipid/protein content) and surface chemistry, with cascading effects on higher trophic levels [29,45]. Third, for biological control, Cd accumulation in adult ACB bodies represents a potential exposure route for natural enemies. Although egg Cd was not significantly elevated, parasitoids may still be affected via host egg quality changes or behavioral modifications, as evidenced by the increased parasitism rates observed in our study [26,28]. While seemingly positive for biocontrol, such stimulation may disrupt host–parasitoid dynamics over multiple generations [27]. These risks collectively demonstrate that a ‘protective’ maternal barrier does not eliminate Cd’s ecological footprint; rather, it redirects selective pressures and consequences to adult stages and interacting species within the food web. This also explains why the basic quality of ACB eggs is not severely damaged by maternal Cd exposure in this experiment.

Critically, despite the high Cd concentrations in female adults, we did not detect a statistically significant increase in Cd concentration in ACB eggs from Cd-exposed mothers compared to controls. This finding suggests that maternal Cd transfer to eggs is limited or highly variable in ACB under our experimental conditions. The large standard error in the egg Cd measurement for the Cd treatment group indicates substantial variability among replicates, which may reflect individual differences in maternal Cd allocation or egg sampling heterogeneity. Limited maternal transfer of Cd to eggs has been observed in other lepidopterans. For example, Cd was transferred to eggs of *C. suppressalis* from Cd-treated larvae, but the concentrations were relatively low compared to earlier life stages [35]. Similarly, Luo et al. [18] found a gradual decrease in Cd concentration from larvae to pupae to adults in ACB, and although they did not measure eggs, their data suggest limited transfer to the next generation. In contrast, studies on other insect orders have shown more pronounced Cd transfer. In the predatory beetle *Serangium japonicum* Chapin (Coleoptera: Coccinellidae), Cd exposure reduced reproductive output and was associated with physiological trade-offs [44], while in the parasitoid *Chouioia cunea* Yang (Hymenoptera: Eulophidae), Cd exposure through the food chain reduced parasitic fitness and induced oxidative damage in offspring [29,30]. The limited maternal Cd transfer in our study may explain why *T. ostriniae* offspring emergence and sex ratio were not negatively affected, as the parasitoid larvae developing inside ACB eggs may have experienced relatively low Cd exposure.

Enhanced parasitism under mild heavy metal stress has been observed in other *Trichogramma* species. For example, Nusillard et al. [46] reported that copper consumed by host larvae had positive effects on the emergence rate and size of emerging *T. cordubensis* parasitoids at the highest copper concentration. Wang et al. [47] demonstrated that a sublethal concentration of the insecticide chlorantraniliprole induced hormesis in *T. japonicum*, enhancing locomotory activity, respiratory metabolism, and mass reproduction. Similarly, Yang et al. [48] found that exposure to a sublethal concentration of spirotetramat accelerated host location and handling behaviors in the parasitoid *Encarsia formosa* Gahan (Hymenoptera: Aphelinidae). These observations support the emerging view that mild chemical stress can stimulate performance in beneficial insects, a phenomenon with important implications for IPM programs that combine chemical and biological control [4,37]. Importantly, the elevated parasitism rate does not necessarily imply enhanced biocontrol efficacy in the field. While a higher number of parasitized eggs may reduce ACB populations in the short term, the long-term consequences of Cd-mediated host alterations on parasitoid population dynamics, including potential transgenerational effects, shifts in sex allocation, or changes in dispersal behavior, remain unexplored [27,28]. Therefore, we emphasize that our findings demonstrate a correlative rather than causal link between maternal Cd exposure and increased parasitism, and that rigorous chemical ecology and behavioral assays are needed to identify the actual cues driving this response.

It is noteworthy that our study used a single Cd concentration (5.0 mg/kg), which is within the sublethal range for ACB [18] and has been shown to produce measurable effects in previous studies. However, the dose-dependent nature of Cd effects on *Trichogramma* parasitoids has been demonstrated in other contexts [27,28]. For example, Wang et al. [27] showed that Cd-contaminated sucrose solutions affected *T. dendrolimi* survival and longevity only at high concentrations, while *T. japonicum* and *T. ostriniae* were more tolerant across a wide concentration range. These studies highlight the importance of using concentration gradients to fully characterize the effects of Cd on parasitoid performance. Future studies should therefore extend our findings by testing multiple Cd concentrations to determine the shape of the dose–response relationship (e.g., linear, threshold, or hormetic) and to identify concentrations that are truly harmless, stimulatory, or harmful to *T. ostriniae*. In contrast to Cd, other heavy metals and pesticides have been shown to negatively affect *Trichogramma* performance [49]. For example, the fungicide Bordeaux mixture (copper-based) reduced the longevity of *T. cordubensis* Vargas and Cabello by 26% upon surface contact, although no oviposition deterrence was observed [50]. The novel insecticides afidopyropen and broflanilide negatively affected emergence rates of *T. evanescens* Westwood and *T. pintoi* Voegele, with broflanilide reducing parasitism performance and walking speed [51]. These studies underscore that *Trichogramma* species vary widely in their sensitivity to different contaminants, emphasizing the need for species-specific risk assessments when designing IPM programs for polluted environments.

Different from the widely reported negative effects of Cd on parasitoid fitness [26,29,52], the most striking finding of our study is that *T. ostriniae* females parasitized significantly more ACB eggs when the host eggs originated from Cd-exposed mothers compared to control eggs. However, this result must be interpreted with caution, as the underlying mechanism remains unclear and the observed increase in parasitism was not accompanied by changes in offspring emergence rate or female progeny ratio. Consequently, we refrain from attributing this effect to any specific mechanism. This stimulatory effect can be explained by the hormesis theory in insect toxicology [36,37]. Notably, this host attractiveness shift is driven by systemic physiological stress of Cd-exposed female ACB, rather than direct Cd accumulation within egg embryos, which explains the non-significant difference in egg Cd concentrations between control and Cd-treated groups. Low-dose or sublethal stress often induces hormetic responses in organisms, and sublethal Cd may change the volatile substances or surface characteristics of ACB eggs, making them more attractive to *T. ostriniae* [14,52]. Wang et al. [45] also found that 20 mg/kg Cd treatment improved the egg quality of *C. cephalonica* and stimulated the reproductive ability of *T. dendrolimi*, which supports our inference that sublethal Cd can indirectly enhance host attractiveness to parasitoids via maternal stress-mediated egg phenotypic modification, instead of egg-internal Cd toxicity. Specifically, sublethal Cd accumulation in female ACB adults disturbs lipid metabolism and stress-related signaling pathways during oogenesis, which remodels two key chemical cues governing parasitoid host foraging. Cadmium-induced oxidative stress upregulates the biosynthesis of low-molecular weight volatile terpenoids and aliphatic aldehydes deposited onto egg surfaces, elevating total volatile emission intensity and altering the relative ratio of attractant semiochemicals; analogous heavy metal-triggered shifts in volatile blends have been validated in maize and lepidopteran hosts, which directly modify olfactory detection by parasitic Hymenoptera [6,9]. Herbivore-induced plant volatiles (HIPVs) and host chemical cues are known to mediate host location and acceptance in *Trichogramma* wasps [20,53], and heavy metals have been shown to influence volatile organic compound (VOC) emissions from plants [14,52]. Cadmium exposure remodels the carbon chain length and saturation ratio of egg cuticular hydrocarbons, the primary contact kairomones for *Trichogramma* host evaluation, generating a chemical profile that elicits stronger antennal chemosensory responses and oviposition triggering in *T. ostriniae* females [20,53]. Though maternal Cd transfer to eggs was not statistically significant in our assay, systemic Cd stress in maternal females reshapes reproductive gland secretions that coat newly laid eggs, independent of direct metal deposition inside egg embryos.

Beyond altered egg chemical cues, we propose sublethal maternal Cd exposure weakens the cellular and humoral immune capacity of ACB eggs. Stress-induced downregulation of egg melanization pathways and antimicrobial peptide synthesis reduces egg defensive responses against parasitoid larval development, increasing maternal acceptance of these hosts by ovipositing wasps [29]. Tan et al. [29] demonstrated that Cd exposure in *Hyphantria cunea* (Drury) (Lepidoptera: Erebidae) pupae reduced cellular and humoral immunity parameters, including hemocyte numbers, melanization activity, and antimicrobial peptide gene expression. Cadmium stress reallocates maternal nutrient reserves to egg biomass (consistent with our observation of heavier Cd-treated female moths), subtly elevating egg nutrient availability for parasitoid progeny. *Trichogramma ostriniae* females exhibit adaptive oviposition preference toward hosts with superior nutritional value, laying more eggs to maximize offspring fitness [16], a phenomenon that could be tested through choice and no-choice behavioral assays [16]. Importantly, the unchanged parasitoid emergence rate and female offspring ratio across treatments rule out severe impairment of egg nutritional suitability, confirming that the stimulatory parasitism effect arises from behavioral host preference rather than compromised offspring survival.

Our findings carry important practical implications. First, the enhanced parasitism and unaltered offspring fitness of *T. ostriniae* under moderate Cd stress suggest that this species is a robust biocontrol candidate for Cd-polluted maize regions where more sensitive natural enemies may fail [27]. Second, our results demonstrate that sublethal heavy metal effects can be stimulatory rather than suppressive at the trophic interaction level, highlighting the need for multi-trophic experimental designs in environmental risk assessment [4,5]. Third, the stage-specific bioaccumulation data we provide enable predictive modeling of Cd flux through the maize–ACB–parasitoid food chain, informing spatially targeted release strategies for *T. ostriniae* in contaminated farmlands [28]. Finally, the maternal Cd barrier that limits transfer to ACB eggs, while protective for herbivore offspring, may create an ecological trap for parasitoids by enhancing egg attractiveness without altering Cd content, a possibility that warrants multi-generational studies to assess cumulative fitness effects on parasitoid populations [28,45]. These implications collectively bridge fundamental ecotoxicology with applied biological control, supporting evidence-based pest management in contaminated agricultural landscapes.

This study has several limitations. First, we tested only one Cd concentration (5.0 mg/kg), which, although ecologically relevant and sublethal for ACB, does not allow characterization of the full dose–response relationship. Future studies should employ concentration gradients (ranging from environmentally realistic to elevated levels) to identify thresholds for stimulation, no effect, and toxicity, and to test for non-monotonic (hormetic) responses [36,37]. Second, our experiments were conducted under controlled laboratory conditions, which do not fully replicate the complexity of field environments where multiple abiotic and biotic stressors interact. Field or semi-field studies are needed to validate our findings under more realistic conditions [24,47]. Given that Cd transfer from herbivores to parasitoids has been documented in other systems [30,42,45], future research should quantify Cd bioaccumulation in *Trichogramma* and assess its effects on parasitoid fitness parameters across multiple generations [28].

## 5. Conclusions

In summary, this study demonstrates that sublethal cadmium exposure at 5.0 mg/kg produces stage-specific effects on the Asian corn borer, significantly increasing female adult body weight without affecting larval or pupal weights, and leads to substantial Cd bioaccumulation in larvae, pupae, and adults, with limited maternal transfer to eggs. More importantly, we found that *T. ostriniae* females parasitized significantly more Cd-contaminated ACB eggs compared to control eggs, without compromising offspring emergence rate or female progeny ratio. This stimulatory effect on parasitism, observed under a single sublethal Cd concentration, highlights the potential for complex, non-linear bottom-up effects of heavy metal pollution on biological control services. Our findings underscore the importance of species-specific and concentration-dependent risk assessments when deploying biological control agents in Cd-contaminated agricultural landscapes. *Trichogramma ostriniae* appears to be a relatively robust biocontrol agent under moderate Cd stress, but further research using concentration gradients, multi-generational exposures, and field conditions is urgently needed to fully characterize the ecological consequences of heavy metal pollution on pest–natural enemy interactions and to guide evidence-based IPM strategies in contaminated regions.

## Figures and Tables

**Table 1:** Cd concentration (mg/kg) in different stages of the Asian corn borer.

Stage	Control (0 mg/kg)	Treatment (5.0 mg/kg)
Larva	0.08 ± 0.01 b	38.07 ± 3.22 a
Pupa (female)	0.11 ± 0.01 b	41.23 ± 2.43 a
Adult (female)	0.04 ± 0.00 b	3.31 ± 0.11 a
Egg	0.05 ± 0.01 a	0.19 ± 0.09 a

Values are given as means ± SE. Significance was determined by the Mann–Whitney *U* test (*p* < 0.05). Different lowercase letters indicate significant differences at *p* < 0.05.

## Data Availability

The original contributions presented in this study are included in the article. Further inquiries can be directed to the corresponding author.
